# Docosahexaenoic Acid Alleviates Brain Damage by Promoting Mitophagy in Mice with Ischaemic Stroke

**DOI:** 10.1155/2022/3119649

**Published:** 2022-10-08

**Authors:** Eryi Sun, Jing Zhang, Yan Deng, Jun Wang, Qi Wu, Wei Chen, Xiaodong Ma, Siyuan Chen, Xin Xiang, Yujie Chen, Tairong Wu, Yang Yang, Bo Chen

**Affiliations:** ^1^Department of Neurosurgery, The Affiliated People's Hospital of Jiangsu University, Zhenjiang 212002, China; ^2^Department of Nursing, The 904th Hospital of PLA, Medical School of Anhui Medical University, Wuxi 214044, China; ^3^West China Hospital of Sichuan University, Chengdu 610041, China; ^4^Department of Neurosurgery, The Affiliated Hospital of Guizhou Medical University, Guiyang 550004, China; ^5^Department of Neurosurgery, Southwest Hospital, Third Military Medical University (Army Medical University), Chongqing 400038, China; ^6^Department of Traditional Chinese Medicine, The Affiliated People's Hospital of Jiangsu University, Zhenjiang 212002, China

## Abstract

Mitophagy, the selective removal of damaged mitochondria through autophagy, is crucial for mitochondrial turnover and quality control. Docosahexaenoic acid (DHA), an essential omega-3 fatty acid, protects mitochondria in various diseases. This study aimed to investigate the neuroprotective role of DHA in ischaemic stroke models *in vitro* and *in vivo* and its involvement in mitophagy and mitochondrial dysfunction. A mouse model of ischaemic stroke was established through middle cerebral artery occlusion (MCAO). To simulate ischaemic stroke *in vitro*, PC12 cells were subjected to oxygen–glucose deprivation (OGD). Immunofluorescence analysis, western blotting (WB), electron microscopy (EM), functional behavioural tests, and Seahorse assay were used for analysis. DHA treatment significantly alleviated the brain infarction volume, neuronal apoptosis, and behavioural dysfunction in mice with ischaemic stroke. In addition, DHA enhanced mitophagy by significantly increasing the number of autophagosomes and LC3-positive mitochondria in neurons. The Seahorse assay revealed that DHA increased glutamate and succinate metabolism in neurons after ischaemic stroke. JC-1 and MitoSox staining, and evaluation of ATP levels indicated that DHA-induced mitophagy alleviated reactive oxygen species (ROS) accumulation and mitochondrial injury. Mechanistically, DHA improved mitochondrial dynamics by increasing the expression of dynamin-related protein 1 (Drp1), LC3, and the mitophagy clearance protein Pink1/Parkin. Mdivi-1, a specific mitophagy inhibitor, abrogated the neuroprotective effects of DHA, indicating that DHA protected neurons by enhancing mitophagy. Therefore, DHA can protect against neuronal apoptosis after stroke by clearing the damaged mitochondria through Pink1/Parkin-mediated mitophagy and by alleviating mitochondrial dysfunction.

## 1. Introduction

Ischaemic stroke occurs when blood supply to a part of the brain is interrupted and is associated with high mortality and disability worldwide [1]. Re-establishment of blood flow is essential for the recovery of ischaemic brain tissues; however, the sudden reperfusion during reconstitution of the blood flow may cause secondary injury [2]. Reperfusion increases the production of reactive oxygen species (ROS) in mitochondria, resulting in calcium influx and the subsequent activation of the caspase-dependent or caspase-independent apoptotic pathway, eventually leading to massive neural death [3]. Modulation of oxidative stress and neuronal apoptosis represents an important therapeutic strategy for ischaemia/reperfusion (I/R) brain injury [4, 5].

Mitochondria are the energy centres of eukaryotic cells and are abundant in metabolically active tissues, including the brain [6]. Mitochondrial dysfunction owing to oxidative stress, energy failure, and disruption of cellular calcium homeostasis is associated with multiple pathological conditions [7, 8]. In addition, an imbalance between mitochondrial fission and fusion can trigger mitochondrial dysfunction [9]. Mitochondrial turnover and quality control rely on a finely tuned balance between mitophagy and mitochondrial biogenesis [10]. Mitophagy is a process in which mitochondria-derived vesicles engulf selected mitochondrial cargoes and deliver them to lysosomes or peroxisomes for degradation [11, 12]. Mitophagy ensures neuronal survival in a physiological state; however, excessive or inadequate levels of mitophagy during pathological conditions can lead to neuronal death in ischaemic stroke [13]. Therefore, novel, effective drugs should be developed for maintaining mitochondrial dynamic homeostasis and promoting neuronal survival after ischaemic stroke.

Docosahexaenoic acid (DHA) is an essential polyunsaturated fatty acid (PUFA) that is abundantly found in cod liver oil and seaweed [14]. Recent studies have shown that DHA promotes neural development [15], reduces neurodegenerative damage [16], delays brain ageing [17], and enhances memory function [18]. The neuroprotective effects of DHA are mediated via inhibition of hyperoxia-induced mitochondrial ROS production [19] and neuroinflammation [20]. This potential mechanism is supported by the significant attenuation of oxidative brain damage in tri-DHA-treated mice subjected to hypoxic injury [21]. Additionally, mitochondria are the targets of intracellular DHA [22, 23]; however, the effects of DHA on mitochondrial function after ischaemic stroke remain unclear. This study aimed to evaluate the neuroprotective effects of DHA using *in vivo* and *in vitro* models of ischaemic stroke and investigate the role and mechanism of action of DHA in mitochondrial dysfunction and mitophagy.

## 2. Materials and Methods

### 2.1. Animals

Adult C57BL/6 mice (age, 6 weeks; weight, 20 ± 1 g) were purchased from the Laboratory Animal Center of Jiangsu University (Zhenjiang, Jiangsu, China) and the Laboratory Animal Center of Third Military Medical University (Chongqing, China). The mice were housed at a constant temperature of 25°C and humidity of 40–60% with a 12/12-h light/dark cycle and were provided food and water *ad libitum*. This study was approved by the Institutional Animal Care and Use Committees of Jiangsu University and Third Military Medical University (number of ethical committee approval: AMUWEC2020761). The study protocol complies with the Animal Research: Reporting in vivo Experiments (ARRIVE) and the National Institutes of Health Guide for the Care and Use of Laboratory Animals guidelines.

### 2.2. Establishment of an Ischaemic Stroke Model

Ischaemic stroke was induced in mice via middle carotid artery occlusion (MCAO) as described in our previous study [24]. Briefly, mice were anaesthetised via intraperitoneal injection of 1% pentobarbital sodium at a dose of 3 mL/kg. The mice were fixed on an operating table in a supine position, and the skin was cleaned with an iodophor disinfectant. An incision was made right of the midline of the neck, and the thyroid junction was separated bluntly to expose the cervical muscle group. The muscles were bluntly dissected to expose the right common carotid artery and vagus nerve, followed by progressive exposure of the common carotid bifurcation, external carotid artery, and internal carotid artery. The distal end of the external carotid artery was ligated with a 6-0 suture, followed by transparent ligation of the proximal end of the common carotid artery and the internal carotid artery. The external carotid artery near the heart was tightened with a loop, and a small opening was made halfway to the distal end of the external artery. The tied line was inserted into the opening, the external artery loop was secured, and the internal artery line was released until the closure at the beginning of the middle cerebral artery, with around the near heart external carotid artery thread fasten wire tied to gently, release the common artery line, layered suture muscle and skin, keep line end position. The bolt was pulled out to restore blood supply to the middle cerebral artery after 1 h. In sham-operated mice, the vessels were separated without inserting a suppository.

### 2.3. Oxygen–Glucose Deprivation Model and Treatment

The PC12 cell line (CRL-1721.1, ATCC, Rockville, MD, USA) is an extensively used cell model in neurobiology because it has some features of mature neurons [25]. In this study, PC12 cells were cultured in Dulbecco's modified Eagle medium supplemented with nutrient mixture F-12 (DMEM/F12) and 10% foetal bovine serum. To establish oxygen–glucose deprivation (OGD) conditions, the cells were incubated with glucose-free Earle's balanced salt solution for 2 h in a hypoxic chamber that was continuously flushed with 95% N_2_ and 5% CO_2_ at 37°C to achieve 0.5% O_2_. The cells were reoxygenated by culturing them in a regular medium with 95% air and 5% CO_2_. Subsequently, the cells were treated with DHA (100 mM) immediately after OGD for 12 h [26].

### 2.4. Study Design and Drug Administration

**Figure 1:**
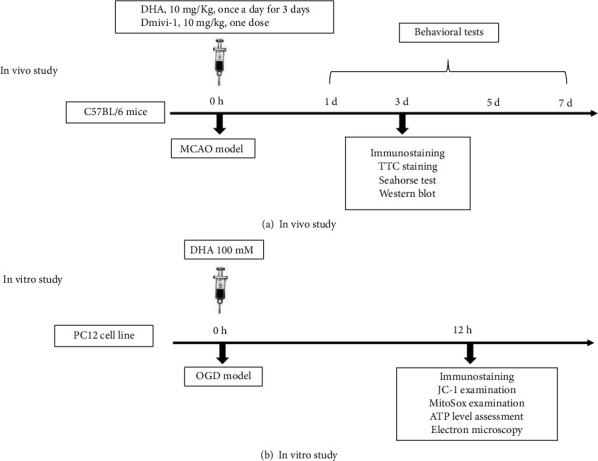
Experimental outline. Schematic diagrams representing in vivo (a) and in vitro (b) experiments.

Experiment 1 (Figure 1(a)): To evaluate the neuroprotective effects of DHA against MCAO, mice were randomly divided into the following groups: sham, MCAO + vehicle (Veh), and MCAO + DHA groups. DHA (10 mg/kg, D2534, Sigma, St. Louis, MO, USA) was dissolved in sterile saline and administered intraperitoneally (i.p.) in mice in the DHA-treated group immediately after reperfusion, and the treatment was repeated once daily for 3 days [20]. Mice in the vehicle and sham groups were administered an equivalent volume (5 mL/kg) of sterile saline via intraperitoneal injection. TTC and TUNEL staining were used to analyse the infarct volume and neuronal death 3 days after MCAO.

Experiment 2 (Figures 1(a) and 1(b)): To investigate whether mitophagy mediated the neuroprotective effects of DHA *in vivo*, mitophagy was evaluated via immunofluorescence staining of LC3B and NeuN. An *in vitro* OGD model was established using PC12 cells to improve the visualisation of mitophagosomes, and the cells were randomly divided into the following groups: control, OGD + Veh, and OGD + DHA groups. Cells in different groups were labelled with LC3B and MitoTracker to evaluate mitophagy, and an electron microscope was used to visualise mitophagosomes. Additionally, mitophagy-associated proteins were analysed via immunoblotting to explore their potential underlying mechanisms.

Experiment 3 (Figures 1(a) and 1(b)): Given that DHA protected mitochondrial function both *in vivo* and *in vitro*, mitochondrial function was examined in the MCAO and OGD models, including the mitochondrial membrane potential, mitochondrial ROS production, ATP content, and mitochondrial metabolism.

Experiment 4 (Figure 1(a)): To verify that the neuroprotective effects of DHA are dependent on mitophagy, Mdivi-1, a selective inhibitor of mitophagy, was used to intervene with DHA-induced mitophagy. Mice were randomly divided into the following groups: sham, MCAO + Veh, MCAO + DHA, and MCAO + DHA + Mdivi-1 groups. For Mdivi-1 (M0199, Sigma) treatment, mice were intraperitoneally administered a single dose of 10-mg/kg Mdivi-1 2 h after reperfusion [27]. TTC and TUNEL staining and behavioural tests were used for further analysis.

### 2.5. TTC Staining and Infarct Measurement

Mice were euthanised 3 days after MCAO, and their brains were removed, frozen at -20°C, and sliced into five 2-mm-thick coronal sections. The sections were stained with 2% 2,3,5-tri-phenyltetrazolium chloride (TTC, T8877, Sigma, St. Louis, MO, USA) at 37°C for 20 min and preserved in 10% phosphate-buffered formalin as described previously [24]. The infarct area was calculated by subtracting the normal area stained with TTC in the ischaemic hemisphere from the area of the non-ischaemic hemisphere. The infarct volume was measured by adding the infarct areas of all sections and multiplying the sum by slice thickness.

### 2.6. Electron Microscopy

Transmission electron microscopy was performed as described previously [28]. Cells stimulated with OGD were centrifuged, and pellets were collected. The pellets were fixed in 2.5% glutaraldehyde, solidified using 1% cooled agarose solution and fixed in 1% OsO4 in PBS for 2 h at room temperature. Thereafter, the blocks were washed thrice with PBS (10 min each time), dehydrated using a graded series of ethanol (30%, 50%, 70%, 80%, and 95% ethanol for 20 min each, and twice in 100% ethanol for 20 min each time), and immersed in acetone (twice for 15 min each time). After infiltration in a 1 : 1 mixture of propyleneoxide and TAAB Epon, the samples were embedded in TAAB Epon and polymerised at 60°C for 48 h. Subsequently, the samples were sectioned (60 nm) using an ultramicrotome (LKB-V, LKB Produkter AB, Bromma, Sweden) and examined using a transmission electron microscope (TECNAI10, Philips, Eindhoven, The Netherlands). Images were captured with an AMT 2 k CCD camera.

### 2.7. Immunofluorescence Analysis

Frozen brain sections were washed thrice with PBS on a shaker (5 min each time) and incubated with 3% BSA for 30 min to block non-specific binding. The sections were incubated with mouse anti-NeuN (1 : 400, MAB377, sigma) and rabbit anti-LC3B (1 : 100, ZRB100, Sigma) antibodies (diluted in blocking buffer) at 4°C overnight. The following day, the sections were incubated with AlexaFluor 555-conjugated goat anti-mouse and AlexaFluor 488-conjugated goat anti-rabbit (1 : 200, Invitrogen, Carlsbad, CA, USA) antibodies for 1 h at room temperature. Thereafter, the sections were washed thrice with PBS, counterstained with DAPI for 10 min at room temperature, washed again, and incubated with a fluorescence quenching reagent for 5 min. NeuN- and LC3B-positive cells were captured and counted in the penumbra area per section, and statistical comparisons were made according to the number of cells per unit area. Autophagosomes and mitochondria in culture cells were stained using the Premo mitophagy sensor LC3B-GFP kit (P36235, Life Technologies) and MitoTracker Deep Red (8778S, Cell Signaling Technology (CST, Boston, MA, USA) according to the manufacturer's instructions. The stained cells and tissues were imaged on a Zeiss LSM 880 Airyscan Confocal Microscope. Z-stack images were acquired using Airyscan detectors and a piezoelectric high-precision stage using the Zen software (Carl Zeiss Microscopy). Images were processed for 3D deconvolution using the Zen software and prepared for publication using the ImageJ software.

### 2.8. Mitochondrial Membrane Potential and Mitochondrial ROS Analyses

The function of mitochondria in PC12 cells in each group was examined as described in a previous study [29]. Mitochondria were labelled with MitoTracker®Green (1 : 1000, 9074, CST), and nuclei were stained with Hoechst 33342 (1 : 1000, 14533, Sigma). Thereafter, 5,5′,6,6 ′ -tetrachloro-1,1 ′ ,3,3 ′ -tetraethylbenzimidazolylcarbocyanine iodide (JC-1, 0.5 *μ*g/mL, C2006, Beyotime, Shanghai, China) was used to measure the mitochondrial membrane potential (ΔΨ*m*). Mitochondrial ROS were evaluated using the MitoSOX™ Red reagent (1 : 1000, M36008, Invitrogen) according to the manufacturer's instructions.

### 2.9. Evaluation of ATP Levels

ATP in tissues around the haematoma and culture cells was measured using an ATP assay kit (ab83355; Abcam) as described previously [30]. Briefly, tissues or cells were washed in cold PBS, homogenised, and centrifuged to collect the supernatant, and the samples were mixed with assay buffer. ATP reaction mixture and background control were added to 96-well plates and incubated for 30 min in the dark. Absorbance was measured using a microplate reader, and the mean optical density was used to estimate the intracellular ATP concentration relative to the standard curve.

### 2.10. Mitochondrial Seahorse Detection

Mitochondria were extracted from the brain tissues using the Mitochondria Extraction Kit (C3606, Beyotime) according to the manufacturer's instructions. Freshly isolated mitochondria (5 *μ*g) were added to Seahorse probe plates (hydrated 6 h before the experiment), and 50 *μ*L of mitochondrial analysis solution (70-mM sucrose, 220-mM mannitol, 2-mM HEPES, 10-mM potassium dihydrogen phosphate, 5-mM magnesium chloride, 1-mM ethylene glycol ditetra-acetic acid, and 0.2% w/v BSA without fatty acid) supplemented with 10-mM succinate or 2-nM glutamate was added to the plates. The samples were centrifuged at 4°C for 20 min at 2000 g. Adenosine diphosphate (ADP), oligomycin (oligo), carbonyl cyanide p-trifluoromethoxy-phenylhydrazone (FCCP), and antimycin A (AA) were added to probe plates A, B, C, and D at the final concentration of 4 mM, 2.5 *μ*g/mL, *4μM* and 4*μ*M, respectively. The detection time and cycle times were set, and the probe plates were placed in the Seahorse XFE24 analyser. After centrifugation, the plates were removed, and 450 *μ*L of the corresponding MAS detection solution containing different substrates was added to each well and incubated in a carbon dioxide-free incubator at 37°C for 10 min. After probe plate correction, the oxygen consumption rate (OCR) (pmol/min) of mitochondria was evaluated.

### 2.11. Western Blotting

Brain tissues around the penumbra were collected using a microscope (RWD Life Science) and lysed in precooled radio immunoprecipitation assay buffer (RAPI) as described previously [31]. Briefly, 50 *μ*g of protein was separated via SDA-PAGE and transferred onto a nitrocellulose membrane. The membrane was blocked with 5% non-fat milk in Tris-buffered saline and Tween 20 (pH 7.6) and incubated overnight with primary antibodies against Drp1 (1 : 2000, 12957-1-AP, Proteintech, Wuhan, China), MFN2 (1 : 1000, 12186-1-AP, Proteintech), LC3 (1 : 1000, ab51520, Abcam, Cambridge, UK), Parkin (1 : 1000, 2132S, CST), Pink1 (1 : 1000, ab23707, Abcam), COX IV (1 : 5000, 4850, CST), TOM20 (1 : 1000, 11802-1-AP, Proteintech), and GAPDH (1 : 5000, 10494-1-AP, Proteintech) at 4°C. Subsequently, the membrane was incubated with appropriate secondary antibodies (CST) at room temperature. An enhanced chemiluminescence reagent kit was used to visualise immunoreactive bands in a blinded manner using the ImageJ software (National Institutes of Health). Five animals from each group were used for analysis, and the experiment was performed in triplicate.

### 2.12. Behavioural Tests

All behavioural tests were performed in a double-blind manner on days 1, 3, 5, and 7 of MCAO as described previously [28].

An 18-point scoring system was used to evaluate neurological deficits, including spontaneous activity (in a cage for 5 min), spontaneous movement of all limbs, movement of the forelimbs (outstretching while the animal was held by the tail), climbing the wall of a wire cage, reaction to touch on both sides of the trunk, and response to having the vibrissae touched.

The beam walking test was performed to assess the subtle loss of fine movement capacity associated with the corticospinal tract. The test evaluates the ability of a mouse to remain upright and walk on a narrow beam with a width of 0.6 cm and length of 120 cm, which is placed 60 cm above the soft bedding material. Mice whose paws slipped down the horizontal surface of the beam (foot faults) fewer than 10 times per 50 steps were selected for further analysis. The number of contralateral forelimb and hindlimb foot faults within 50 steps was counted, and mice that took fewer than 50 steps after MCAO were excluded.

The rotarod test instrument was debugged and adjusted to low speed, and mice were preconditioned for 1 min. The initial and maximum speeds ranged from 5 to 35 rpms. The falling latency of mice was recorded automatically over 5 min, with a maximum score of 300 s. The test was performed thrice at 10-min intervals, and the average value was calculated. All mice were trained 1 day before surgery, and those with a latency period of <250 s were excluded.

### 2.13. Statistical Analysis

The evaluator was blinded to the grouping of mice. All data were analysed using the SPSS Statistics (version 19.0) (IBM, Armonk, NY, USA) and GraphPad Prism (version 8.0) (GraphPad Software Inc., San Diego, CA, USA) software. The two-tailed Student's *t*-test was used for comparing data between two groups. One-way analysis of variance followed by Tukey's post hoc test was used for comparing data among more than two groups. Two-way analysis of variance followed by Tukey's post hoc test was used for comparing the results of behavioural tests. Error bars in all figures represent the mean ± standard error of the mean (SEM). A *P*-value of <0.05 was considered significant.

## 3. Results

### 3.1. DHA Ameliorates Brain Infarction and Neuronal Death Induced by MCAO

**Figure 2:**
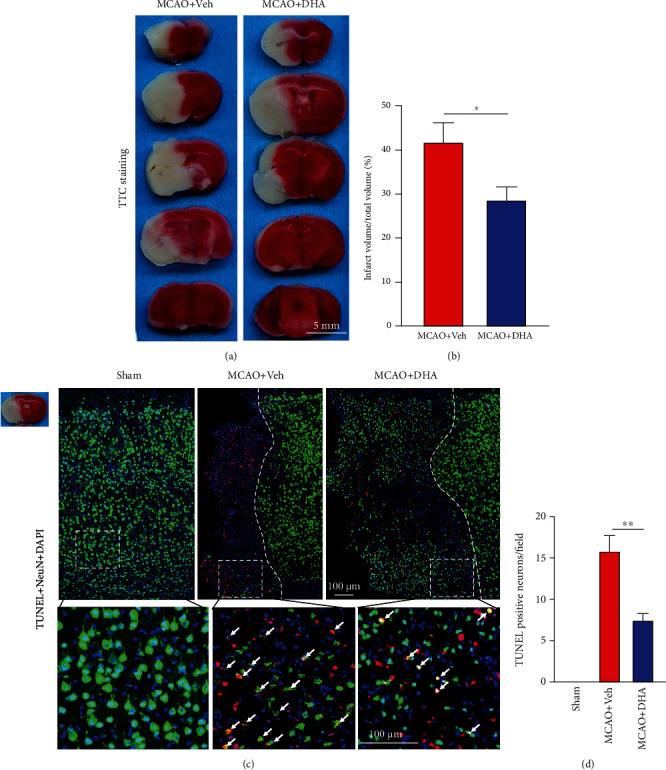
DHA reduced the infarct volume and alleviated neuronal death after ischaemic stroke. (a) Representative images of TTC-stained brain sections in each group (scale bar =5 mm). (b) Quantification of the infarct volume in each group. (c) Representative fluorescence images of cells co-labelled with TUNEL and NeuN in the ischaemic penumbra in each group (scale bar =100 *μ*m). White dotted lines separate the normal and penumbra regions, and white dotted squares demarcate the enlarged area. White arrows indicate neurons co-labelled with TUNEL and NeuN. (d) Number of TUNEL-positive neurons per field in the infarct area. Data are expressed as the mean ± SEM (*n* =6 animals from each group) (^∗^, *P* < 0.05; ^∗∗^, *P* < 0.01). The two-tailed Student's *t*-test (b) and one-way ANOVA followed by Tukey's post hoc test (d) were used for data comparison.

To examine the effects of DHA on cerebral I/R injury, mouse models of MCAO were established and treated with DHA. TTC staining showed that the infarct volume was significantly smaller after 3 days of MCAO in DHA-treated mice than in vehicle-treated mice (*P* < 0.05, Figures 2(a) and 2(b)). In addition, NeuN (green) and TUNEL (red) double staining revealed DHA treatment significantly reduced the number of dead neurons in the penumbra (*P* < 0.01, Figures 2(c) and 2(d)).

### 3.2. DHA Enhanced Neuronal Mitophagy in the MCAO and OGD Models

**Figure 3:**
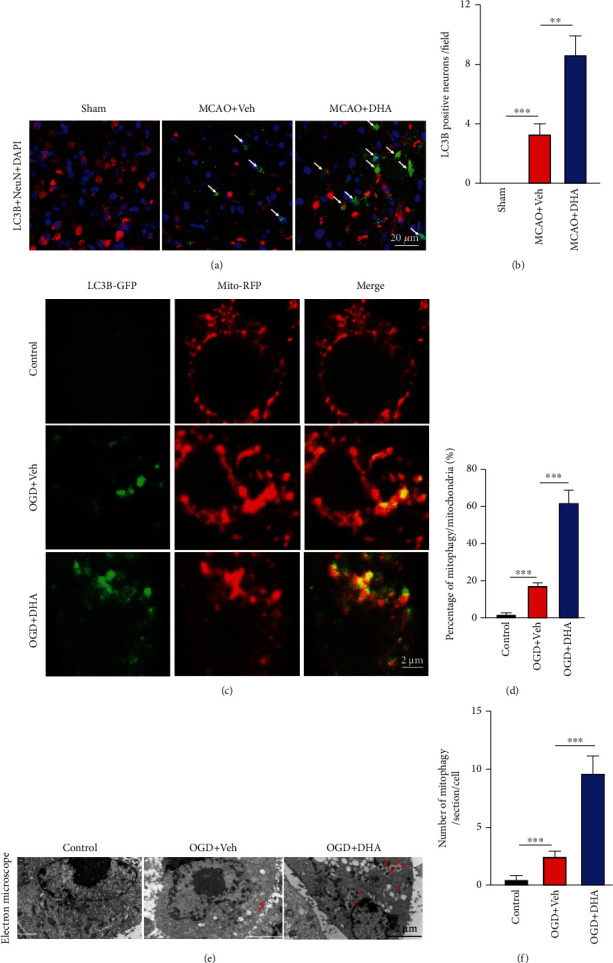
DHA promoted neuronal mitophagy in the MCAO and OGD models. (a) Representative fluorescence images demonstrating co-localisation of LC3B (green) and NeuN (red) in the ischaemic penumbra in each group (scale bar =20 *μ*m). White arrows indicate LC3B-positive neurons. (b) Number of LC3B-positive neurons per field in each group. (c) Representative fluorescence images demonstrating LC3B (green) and MitoTracker (red) staining in PC12 cells in each group (scale bar = 2 *μ*m). (d) Proportion of LC3B-positive mitochondria in each group. (e) Representative electron microscopy images demonstrating mitophagosomes (red arrows) in PC12 cells in each group (scale bar = 2 *μ*m). (f) Number of mitophagosomes per section per cell in each group. Data are expressed as mean ± SEM (*n* =6 animals [a–b, *in vivo*] or 5 independent cell cultures [c–f, *in vitro*] for each group) (^∗∗^, *P* < 0.01; ^∗∗∗^, *P* < 0.001). One-way ANOVA followed by Tukey's post hoc test was used for data comparison.

Autophagy is vital for neuronal homeostasis and function [32]. Evidence suggests that autophagy is impaired during cerebral ischaemia, leading to neuronal dysfunction and neurodegeneration [33, 34]. To investigate whether DHA protects neurons by activating autophagy, immunofluorescence staining for LC3, a reliable marker for monitoring autophagy, was performed, and neurons were co-labelled with NeuN. As shown in Figure 3(a), although several LC3-positive neurons were observed in the penumbra after MCAO, their number was significantly higher in the DHA-treated group than in the MCAO + Veh group (*P* < 0.01, Figures 3(a) and 3(b)), indicating that DHA promoted neuronal autophagy after stroke.

Recent studies have reported that mitophagy plays an important role in maintaining neuronal survival [11]. Therefore, in this study, immunofluorescence staining and EM were used to observe structural changes in mitochondria in an OGD model of PC12 cells. To visualise mitophagy, PC12 cells were transduced with the LC3B-GFP sensor to observe autophagy and co-labelled with MitoTracker Deep Red to visualise the mitochondria. After 2 h of OGD stimulation, 17.33% of mitochondria were co-labelled with LC3B and MitoTracker in the OGD + Veh group, indicating the formation of mitophagosomes in PC12 cells after OGD. However, approximately 62.05% of mitochondria were co-labelled with LC3B and MitoTracker in the OGD + DHA group (*P* < 0.001, Figures 3(c) and 3(d)). Furthermore, EM showed that a few mitochondria were engulfed by a double-membrane structure (red arrows in Figure 3(e)) in the OGD + Veh group, indicating the presence of mitophagy. The number of mitophagosomes was higher in the DHA-treated group than in the OGD + Veh group (*P* < 0.001, Figures 3(e) and 3(f)), suggesting that DHA enhances OGD-induced mitophagy.

### 3.3. DHA Protected Mitochondrial Function in the MCAO and OGD Models

Mitophagy-mediated clearance of damaged mitochondria is essential for mitochondrial function and cellular homeostasis [12]. In this study, the function of mitochondria was examined *in vivo* and *in vitro* using the MCAO and OGD models, respectively, including the evaluation of ΔΨ*m*, mitochondrial ROS production, ATP content, and mitochondrial metabolism.

**Figure 4:**
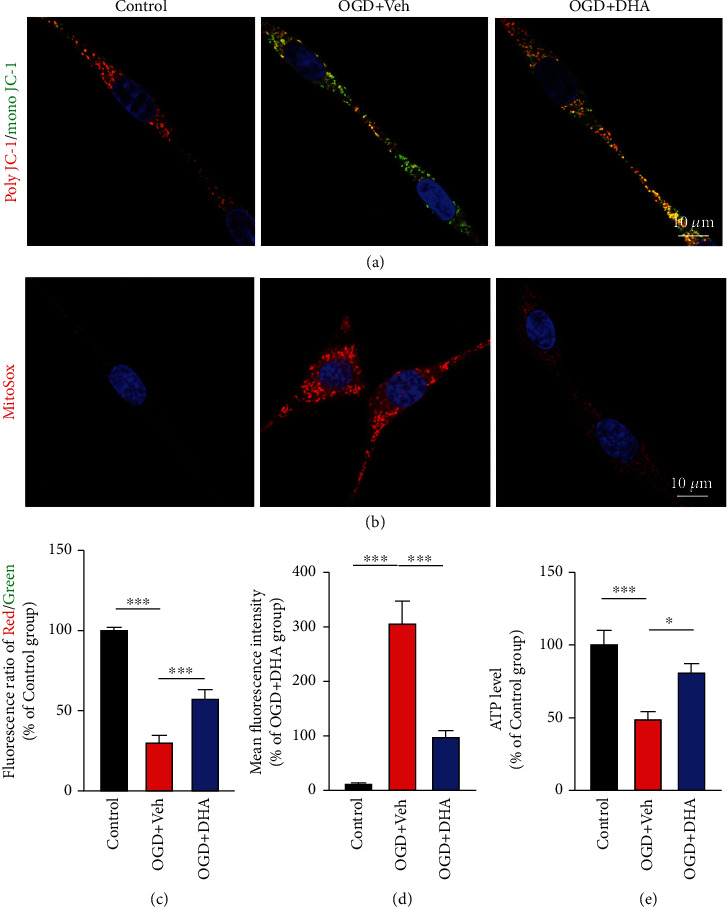
DHA preserved mitochondrial function in the OGD model. (a) Representative images of poly JC-1 (red), mono JC-1 (green), and Hoechst (blue) staining for the evaluation of mitochondrial membrane potential and nuclei in each group (scale bar = 10 *μ*m). (b) Representative images of MitoSox (red) and Hoechst (blue) staining for the evaluation of ROS levels and nuclei in each group (scale bar = 10 *μ*m). (c) Quantification of the poly JC-1/mono JC-1 fluorescence ratio in each group (% of the control group). (d) Quantification of the mean fluorescence intensity in each group (% of the OGD + DHA group). (e) Quantification of ATP levels in each group (% of the control group). Data are expressed as mean ± SEM (*n* =5 independent cell cultures or each group) (^∗^, *P* < 0.05; ^∗∗∗^, *P* < 0.001). One-way ANOVA followed by Tukey's post hoc test was used for data comparison.

ΔΨ*m* generated by mitochondrial proton pumps (complexes I, III, and IV) is an essential component in energy storage during oxidative phosphorylation, and its dissipation is a hallmark of mitochondrial dysfunction [35]. Live-cell imaging with JC-1 was performed to evaluate ΔΨ*m*. Poly JC-1 is red in colour and labels mitochondria with high ΔΨ*m*, whereas mono JC-1 is green in colour and labels mitochondria with low ΔΨ*m*. Changes in ΔΨ*m* were expressed as changes in the poly JC-1 (red)/mono JC-1 (green) fluorescence ratio. Moreover, a decreased TMRM signal indicated low ΔΨ*m*. The results revealed that the staining intensity of mono JC-1 was increased and the poly JC-1/mono JC-1 fluorescence ratio was significantly decreased in the OGD + Veh group (*P* < 0.001; Figures 4(a) and 4(c)). However, the poly JC-1/mono JC-1 fluorescence ratio was significantly higher in the OGD + DHA group than in the OGD + Veh group (*P* < 0.001; Figures 4(a) and 4(c)). Furthermore, to examine the regulatory effects of DHA-mediated mitophagy on mitochondrial ROS, PC12 cells in each group were incubated with MitoSox to label mitochondrial superoxide. As shown in Figure 4(b), mitochondrial ROS levels were higher in the OGD + Veh group than in the control group; however, the levels were significantly decreased after DHA treatment (*P* < 0.001; Figures 4(b) and 4(d)). In addition, ATP levels were significantly lower in the OGD + Veh group than in the control group (*P* < 0.001; Figure 4(e)) but were significantly higher in the OGD + DHA group than in the OGD + Veh group (*P* < 0.05; Figure 4(e)).

**Figure 5:**
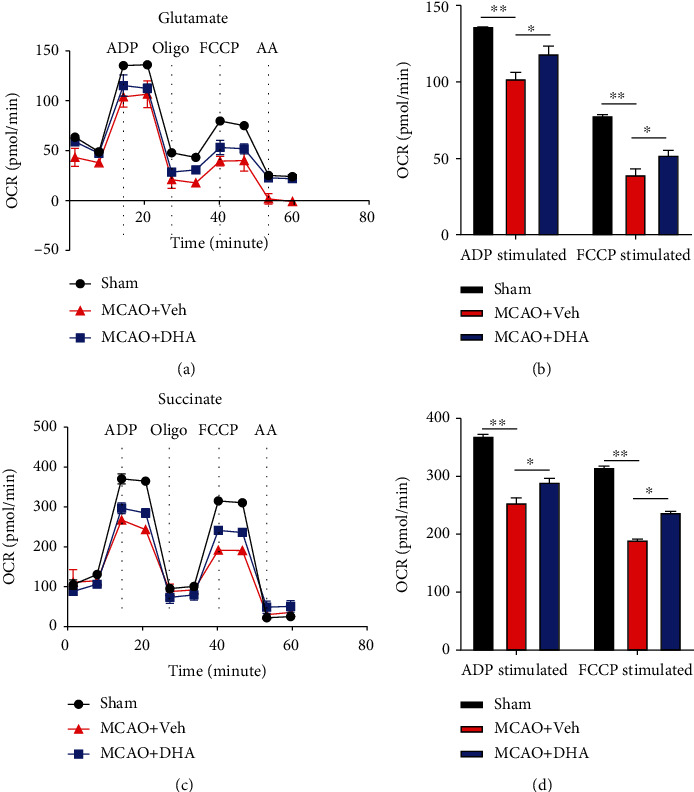
DHA increased mitochondrial metabolism in the MCAO model. (a) The OCR of mitochondria isolated from the penumbra was measured under glutamate stimulation and in response to treatment with the indicated drugs (4-mM ADP, 2.5-*μ*g/mL oligo, 4-*μ*M FCCP, and 4-*μ*M AA) in each group. (b) The OCR of ADP- and FCCP-stimulated mitochondrial respiration in each group. (c) The OCR of mitochondria isolated from the penumbra was measured under succinate stimulation and in response to treatment with the indicated drugs (4-mM ADP, 2.5-*μ*g/mL oligo, 4-*μ*M FCCP, and 4-*μ*M AA) in each group. (d) The OCR of ADP- and FCCP-stimulated mitochondrial respiration in each group. Data are expressed as mean ± SEM (*n* =6 animals from each group) (^∗^, *P* < 0.05; ^∗∗^, *P* < 0.01). One-way ANOVA followed by Tukey's *post hoc* test was used for data comparison. OCR: oxygen consumption rate; ADP: adenosine diphosphate; Oligo: oligomycin; FCCP: carbonyl cyanide p-trifluoromethoxy-phenylhydrazone; AA: antimycin A.

To examine mitochondrial metabolism, mitochondrial respiratory function was measured via the Seahorse assay using different substrates. Mitochondria were isolated from the penumbra in different groups. OCR (pmol/min) during glutamate-driven complex I respiration in the presence of ADP was reduced in the MCAO + Veh group but was significantly improved in the MCAO + DHA group (*P* < 0.05, Figures 5(a) and 5(b)). Furthermore, OCR during FCCP-driven mitochondrial respiration was reduced in the MCAO + Veh group but was significantly restored in the MCAO + DHA group (*P* < 0.05, Figures 5(a) and 5(b)). Similar results were observed for succinate-initiated complex II respiration. OCR during ADP-stimulated state III respiration and FCCP-stimulated maximum respiratory capacity was significantly improved in the MCAO + DHA group compared with the MCAO + Veh group (*P* < 0.05, Figures 5(c) and 5(d)). Altogether, these results indicate that DHA enhances the metabolic capacity of mitochondria after stroke.

### 3.4. Pink1/Parkin-Mediated Mitophagy Mediated the Neuroprotection Function of DHA

PTEN-induced kinase 1 (Pink1) and the E3 ubiquitin ligase Parkin are involved in a highly conserved mitochondrial quality control pathway found in almost every cell type, including neurons [36]. Mitochondrial damage-induced activation of Pink1 stimulates phosphorylation-dependent activation of Parkin and ubiquitin-dependent elimination of mitochondria via mitophagy [37]. To investigate whether the Pink1/Parkin pathway mediated DHA-induced mitophagy, the expression of mitophagy-related proteins was assessed via western blotting.

**Figure 6:**
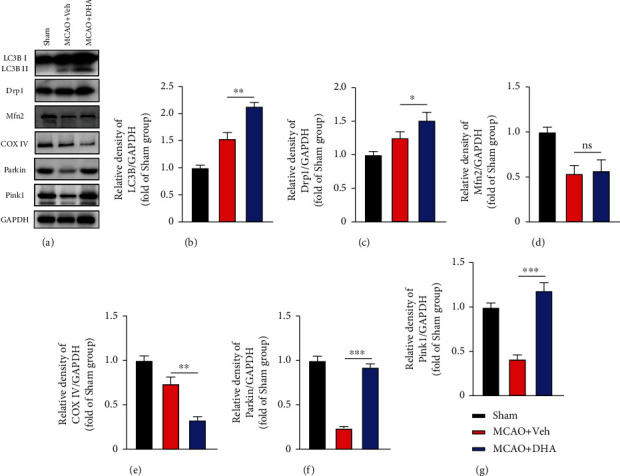
DHA activated mitophagy through the Pink1/Parkin pathway after MCAO. (a) Representative immunoblot bands of mitophagy-related proteins in each group after 3 days of MCAO, including LC3B, Drp1, Mfn2, COX IV, Parkin, Pink1, and GAPDH. (b–g) Quantification of optical density (fold of the sham group) of proteins in each group, including LC3B (b), Drp1 (c), Mfn2 (d), COX IV (e), Parkin (f), and Pink1 (g). Data are expressed as mean ± SEM (*n* =5 animals from each group) (^∗^, *P* < 0.05; ^∗∗^, *P* < 0.01; ^∗∗∗^, *P* < 0.001; ns: not significant). One-way ANOVA followed by Tukey's *post hoc* test was used for data comparison.

LC3 expression was higher in the MCAO + DHA group than in the MCAO + Veh group (*P* < 0.01, Figures 6(a) and 6(b)), suggesting DHA enhanced autophagy after stroke. On analysing the expression of mitochondrial dynamin-related protein 1 (Drp1) and mitofusin 2 (Mfn2), which are the markers of mitochondrial fission and fusion, respectively, Drp1 expression was found to be higher in the MCAO + DHA group than in the MCAO + Veh group (*P* < 0.05, Figures 6(a) and 6(c)). These results indicate that DHA facilitates mitochondrial fission and clearance of damaged mitochondrial segments. Mfn2 expression was lower in the MCAO + Veh group than in the MCAO + DHA group; however, the difference was not significant (*P* > 0.05, Figures 6(a) and 6(d)). COX IV is located in the inner mitochondrial membrane, and its expression indirectly reflects the number of mitochondria and indicates changes in mitophagy. In this study, the expression of COX IV in the MCAO + DHA group was lower compared with that in the MCAO + Veh group (*P* < 0.01, Figures 6(a) and 6(e)), indicating that activation of autophagy was enhanced after DHA treatment. In addition, the expression of Pink1 and Parkin in the brain tissues was significantly lower in the MCAO + DHA group than in the sham group (Figures 6(a), 6(f), and 6(g)). However, DHA treatment significantly restored the expression of both proteins (*P* < 0.001, Figures 6(a), 6(f), and 6(g)). These results indicate that DHA increases Pink1/Parkin-mediated mitophagy to clear damaged mitochondria after MCAO.

### 3.5. Mdivi-1 Abolished the Neuroprotective Effects of DHA after Ischaemic Stroke

To verify that the neuroprotective effects of DHA are dependent on mitophagy, Mdivi-1, a selective inhibitor of mitophagy activation, was used to intervene with DHA-induced mitophagy [27].

**Figure 7:**
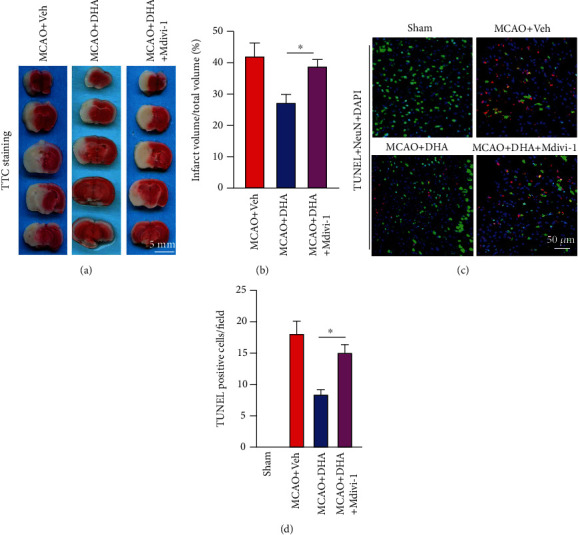
Mdivi-1 abolished the protective effects of DHA after MCAO. (a) Representative images of TTC-stained brain sections in each group (scale bar =5 mm). (b) Quantification of the infarct volume in each group. (c) Representative fluorescence images of cells co-labelled with TUNEL and NeuN in the ischaemic penumbra in each group (scale bar =50 *μ*m). (d) Number of TUNEL-positive neurons per field in the penumbra. Data are expressed as mean ± SEM (*n* =6 animals from each group) (^∗^, *P* < 0.05). One-way ANOVA followed by Tukey's *post hoc* test was used for data comparison.

TTC staining revealed that DHA and Mdivi-1 co-treatment abrogated the protective effects of DHA on the brain infarct volume. The infarct volume was significantly higher in the MCAO + DHA + Mdivi-1 group than in the MCAO + DHA group (*P* < 0.05, Figures 7(a) and 7(b)). In addition, the number of NeuN- and TUNEL-double-positive apoptotic neurons in the penumbra was significantly higher in the MCAO + DHA + Mdivi-1 group than in the MCAO + DHA group (*P* < 0.05, Figures 7(c) and 7(d)).

**Figure 8:**
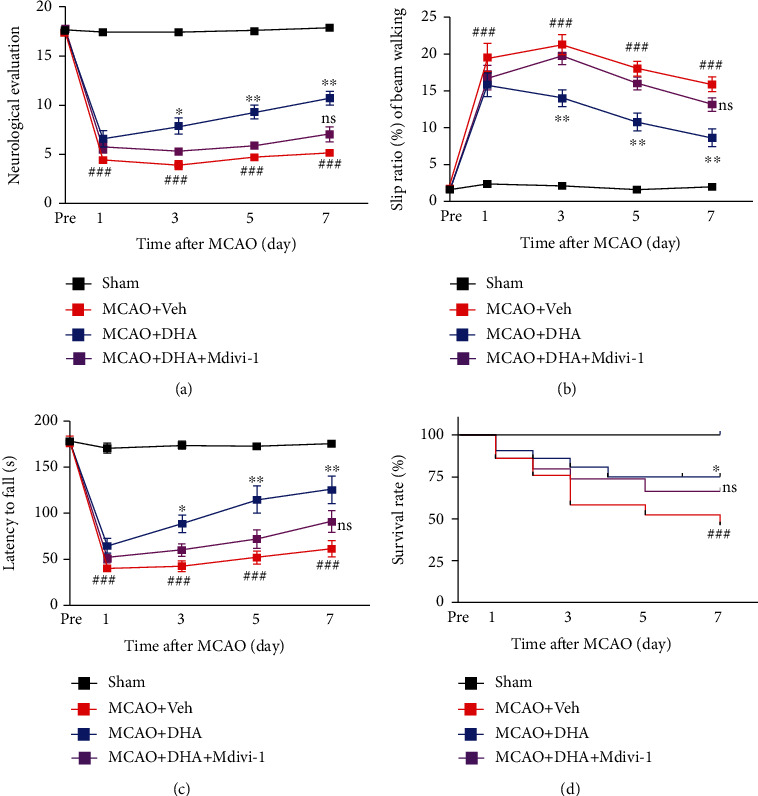
DHA improved neurological behaviour after cerebral I/R injury and its effects were neutralised by Mdivi-1. (a) General movement function was analysed via neurological evaluation (18-point scale) in each group on days 1, 3, and 7 days of MCAO. (b) Quantification of slip frequency (%) of the contralateral limbs within 50 steps in the beam walking test in each group on days 1, 3, and 7 of MCAO. (c) Quantification of latency to fall (s) in the rotarod test in each group on days 1, 3, and 7 of MCAO. (d) Quantification of survival rate (%) in each group on days 1, 3, and 7 of MCAO. Data are expressed as mean ± SEM (*n* = 8 animals from each group) (^**###**^, *P* < 0.001 for the sham group versus the MCAO + Veh group; ^∗^, *P* < 0.05; ^∗∗^, *P* < 0.01; ^∗∗∗^, *P* < 0.001 for the MCAO + Veh group versus the MCAO + DHA group; ns: not significant for the MCAO + DHA group versus the MCAO + DHA + Mdivi-1 group on day 7 of MCAO). Two-way ANOVA followed by Tukey's *post hoc* test was used for data comparison.

Furthermore, neurobehavioural tests were performed on days 1, 3, 5, and 7 of MCAO. There was a significant deficit in general neurological function, more slip steps on beam walking, shorter latency to fall in the rotarod test, and higher survival rate on days 1, 3, 5, and 7 days of MCAO compared with the sham group (*P* < 0.001, Figures 8(a)–8(d)). Compared with mice in the MCAO + Veh group, those in the MCAO + DHA group showed significantly improved performance on neurological evaluation (*P* < 0.05 on day 3 of MCAO; *P* < 0.01 on days 5 and 7 of MCAO; Figure 8(a)), lower slip frequency during beam walking (*P* < 0.01 on days 3, 5, and 7 of MCAO; Figure 8(b)), shorter latency to fall in the rotarod test (*P* < 0.05 on day 3 of MCAO; *P* < 0.01 on days 5 and 7 of MCAO; Figure 8(c)), and improved survival rate (*P* < 0.01 on day 7 of MCAO; Figure 8(d)). However, Mdivi-1 eliminated the protective effects of DHA, and no differences in neurological deficits were observed between the MCAO + DHA + Mdivi-1 and MCAO + Veh groups (*P* > 0.05, Figures 8(a)–8(d)). Altogether, these results indicate that DHA treatment can improve motor function (improved fine motor and grasp abilities) and survival rate after MCAO by enhancing mitophagy, and inhibition of mitophagy can partially neutralise the beneficial effects of DHA.

## 4. Discussion

Mitochondrial dysfunction plays an important role in the pathogenesis of stroke [13]. Calcium overload, opening of the mitochondrial permeability transition pore (mPTP), and excessive ROS production contribute to pathological changes in the mitochondria and neuronal death after stroke [38]. Excessive ROS production further triggers various apoptotic pathways, such as caspase 3-mediated apoptosis, leading to an overwhelming apoptotic positive feedback [39]. In this study, the function of mitochondria and ROS levels were examined both *in vivo* and *in vitro*. MitoSox was used to examine ROS levels in living cells. The fluorescence intensity of MitoSox was significantly increased after OGD, suggesting excessive ROS accumulation. In addition, mitochondrial dysfunction was reflected by decreased ΔΨ*m* and ATP exhaustion. Functionally, respirometry is the gold-standard method for measuring mitochondrial oxygen consumption during respiration because it reflects the activity of electron transport chain complexes [40]. Mitochondrial OCR during ADP-stimulated state III respiration and FCCP-stimulated maximum respiratory capacity was significantly decreased after I/R injury. These results indicate that I/R injury triggers excessive ROS production and mitochondrial impairment.

The dynamic self-regulatory mechanisms of mitochondria, including fusion, fission, and selective degradation, are important for mitochondrial quality control [12]. Mitophagy is a type of self-regulatory mechanism in which damaged or dysfunctional mitochondria are specifically degraded to prevent excessive ROS production and neural cell death [11]. In this study, mitophagy was reflected by the increased number of LC3B-positive neurons in the penumbra *in vivo* and the increased number of LC3B-positive mitochondria and mitophagosomes in PC12 cells after OGD. Mechanistically, the expression of LC3B (an autophagy marker) and Drp1 (a mitochondrial fission marker) was increased, whereas that of Mfn2 (a mitochondrial fusion marker) and COX IV (a mitophagy activation marker) was decreased after stroke. However, the efficiency of stroke-induced mitophagy was low.

DHA is an omega-3 fatty acid that is essential for normal brain growth and cognitive function [15]. Consistent with its importance in the brain, DHA is incepted into the brain via major facilitator superfamily domain-containing protein 2a (Mfsd2a) and is highly enriched in brain phospholipids [41]. During neurotransmission and after brain injury, DHA is released from membrane phospholipids and converted to bioactive mediators, which regulate important signalling pathways associated with membrane integrity, cell survival, synaptogenesis, neuronal excitability, and neuroinflammation. In addition, DHA exerts anti-apoptotic and antioxidant effects by enhancing mitochondrial function in various neural diseases, including cerebral infarction [42, 43]. However, its regulatory roles in mitochondria and the underlying mechanisms remain to be elucidated. In this study, DHA treatment promoted mitophagy in neurons after stroke both *in vivo* and *in vitro*, with an increased number of LC3B-positive neurons in the penumbra and the significantly increased number of LC3B-positive mitochondria and mitophagosomes in PC12 cells after OGD. In addition, the expression of LC3B and Drp1 was increased, whereas that of Mfn2 and COX IV was decreased after DHA treatment, indicating that DHA activates mitophagy. Additionally, DHA treatment alleviated mitochondrial dysfunction by increasing ΔΨ*m* and ATP content and decreasing ROS accumulation and increased the OCR during ADP-stimulated state III respiration and FCCP-stimulated maximum respiratory capacity after I/R injury. Importantly, DHA treatment alleviated motor dysfunction and increased the survival rate after I/R injury. However, the mitophagy inhibitor Mdivi-1 partially abrogated the protective effects of DHA by inhibiting mitophagy.

Pink1/Parkin-mediated mitophagy selectively removes damaged mitochondria [44]. Pink1 and Parkin accumulate in the damaged mitochondria, promote their segregation from the mitochondrial network, and target these organelles for autophagic degradation [45]. Pink1 stabilisation leads to phosphorylation of pre-existing ubiquitin molecules on the mitochondrial surface, which recruit and activate Parkin. The binding of Parkin to the phosphorylated ubiquitin leads to the conjugation of ubiquitin with various substrates and the formation of ubiquitin chains [46]. This study showed that DHA can enhance mitophagy by activating the Pink1/Parkin pathway to clear damaged mitochondria after MCAO.

However, this study has certain limitations: (1) The OGD model was established using PC12 cells; therefore, the results of *in vitro* experiments should be verified using primary culture neurons or *in vivo* experiments. (2) Mitochondria isolated from the penumbra for the Seahorse assay belonged to not only neurons but also non-neuronal cells in the brain. In the future, other cell types should be analysed to elucidate the potential mechanism of action of DHA. (3) This study had a pre-clinical design and hence lacks evidence that DHA can treat ischaemic stroke in clinical settings. Because DHA is currently easily available and used by many elderly patients, prospective clinical studies may yield more convincing results to verify the neuroprotective effects of DHA.

**Figure 9:**
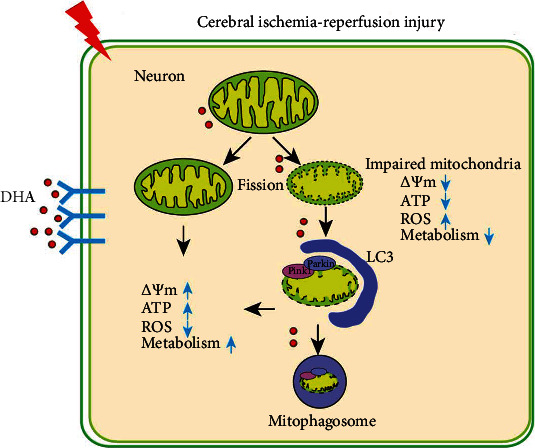
Schematic summary of the study. DHA alleviates mitochondrial dysfunction and neuronal injury after cerebral ischaemia–reperfusion injury by clearing damaged mitochondria via Pink1/Parkin-mediated mitophagy.

This study suggests that DHA promotes mitophagy and clears damaged mitochondria by regulating the Pink1/Parkin pathway (Figure 9). Therefore, this translational drug may be effective for ameliorating the pathophysiology of ischaemic stroke. In addition, maintaining mitochondrial turnover and reducing mitochondrial ROS accumulation may be a key to improving the prognosis of ischaemic stroke.

## 5. Conclusion

DHA alleviates neuronal injury and motor dysfunction after ischaemic stroke by clearing damaged mitochondria via Pink1/Parkin-mediated mitophagy.

## Data Availability

Some or all data, models, or code that support the findings of this study are available from the corresponding author upon reasonable request (list items).
